# Pregnancy Denial: Toward a New Understanding of the Underlying Mechanisms

**DOI:** 10.1007/s11920-023-01448-2

**Published:** 2023-08-19

**Authors:** Natalia Chechko, Elena Losse, Susanne Nehls

**Affiliations:** 1 Department of Psychiatry, Psychotherapy and Psychosomatics, Faculty of Medicine, RWTH Aachen, Pauwelsstraße 30, 52074 Aachen, Germany; 2 Institute of Neuroscience and Medicine: JARA-Institute Brain Structure Function Relationship (INM 10), Research Center Jülich, Jülich, Germany; 3 Institute of Neuroscience and Medicine, Brain & Behavior (INM-7), Research Center Jülich, Wilhelm-Johnen-Strasse, 52428 Jülich, Germany

**Keywords:** Pregnancy denial, Risk factor, Psychopathology

## Abstract

**Purpose of Review:**

Pregnancy denial is the lack of awareness of being pregnant. The aim of the review is to understand why the affected women do not recognize the signs of pregnancy.

**Recent Findings:**

Twelve case reports of pregnancy denial were published in the last ten years. While in five cases the women had an underlying mental disorder, the rest of the cases involved women who either exhibited no physical symptoms or perceived themselves to be not pregnant despite the symptoms (i.e., repression mechanisms).

**Summary:**

Pregnancy denial is considered to be a pathological issue, a likely consequence of trauma, the wish to not have a child, or a psychiatric problem. However, it appears that the majority of cases cannot be linked to any of the above reasons. We argue, therefore, that, in most cases, pregnancy denial is not associated with mental or physiological problems. Under certain circumstances, it can affect any woman.

## Introduction

The acceptance of the state of being pregnant, the attachment to the fetus, preparatory activities, and a realistic perception of the newborn are all part of normal adjustments to pregnancy [1]. Some women, however, remain unaware of being pregnant after 20 weeks of gestation, and sometimes even until delivery.

Known as pregnancy denial, this is not a rare phenomenon. More common than, for instance, rhesus hemolysis, or uterine rupture [2], pregnancy denial after the 20th week of gestation has a frequency of 1:300–516 pregnancies in epidemiological and non-epidemiological studies across Europe and the USA [2–4, 5••, 2, 6, 7], making it more common than triplets [2].

In addition to the time-based criterion for the resolution of pregnancy denial, Miller [8] and Friedman et al. [4] have proposed circumstantial criteria based on a qualitative classification. In what they refer to as affective denial, the pregnancy is acknowledged intellectually, but its emotional significance is downplayed in such a way that the pregnant woman continues her life as though she were not pregnant. In pervasive denial, on the other hand, the woman does not acknowledge her pregnancy either emotionally or intellectually. Psychotic denial leads women to misinterpret the relevant physical changes in a delusional manner, perceiving them as blood clot, some form of cancer, or loose organs. Often surfacing and disappearing during pregnancy, this form of denial may be preceded or exacerbated by identifiable stressors. Typically, women with psychotic denial have a comorbid psychiatric illness and tend not to conceal their pregnancies. Also, people around them are unlikely to be complicit in their denial [8, 9].

The denial of pregnancy can be considered an emotion-driven strategy in the face of circumstances perceived as unalterable [8], with avoidance rather than acknowledgment being seen as the more viable option [10]. For those with cultural or family stigmatization of sexuality, or with a history of sexual trauma, embracing the reality of pregnancy can be difficult [8]. Although pregnancy denial and subsequent neonaticide resulting from the experience of trauma such as incest, rape, or even early trauma, have long been under the spotlight [11, 12], such cases appear to be quite rare. In epidemiological and non-epidemiological studies related to pregnancy denial, trauma histories are either not reported at all or are present only minimally [4, 7, 13••, 14]. According to Delong et al. [13••], women with pregnancy denial are more likely to have had a psychiatric history (23% of women with pregnancy denial reported to have a psychiatric history compared to 8% of their counterparts in the control group). However, the majority of women were mentally healthy. Thus, pregnancy denial is not as closely associated with trauma or other psychiatric conditions as it is assumed to be. The other relevant risk factors are relatively young age, low level of education, precarious work situation, and being single [13••]. However, none of these factors appears specific enough to explain such a dramatic event as pregnancy denial.

The aim of this review was to fully understand the heterogeneity of the phenomenon. In addition, we sought to explore why the affected women, who may be otherwise healthy, do not recognize the signs of being pregnant. What exactly may underlie this lack of pregnancy awareness? To address this question, we collated information from case reports published over the past 10 years, outlining the circumstances prior to and during the denial, as well as the reported reasons thereof: psychiatric status, pregnancy due to a traumatic experience, possible conflict situations and the benefits of assumed non-pregnancy, denial due to personality, and denial as a common defense mechanism in life.

Peer-reviewed and published articles related to key terms around “pregnancy denial,” “cryptic pregnancy,” “stealth pregnancy,” and “unperceived pregnancy” were retrieved from major databases such as PubMed, Web of Science, and Google Scholar. The reference lists of relevant articles were also scanned for additional material pertaining to similarities in symptom presentation across demographic profiles. All seminal research from 2013 to current publications in 2023 was reviewed. Papers were excluded if they were not available in English or if they did not contribute to identifying consistencies in the clinical presentations of pregnancy denial. The included articles are listed in Table 1.

**Table 1:** Overview of the included cases

Reference	Woman’s age	Number of children	Relationship	Psychiatric/psychological traits	Contributing Factors	Traumatic events/stressors	Pregnancy characteristics	Pregnancy detection	Proposed explanation for denial
Chase et al. [9]	33	1	n.a	Major Depressive Disorder	Discontinued duloxetine, late onset of denial	None	Uncomplicated, symptoms integrated into psychosis	Early in pregnancy	Psychiatric Condition
Dua and Grover [16]	25	n.a	Married	History since age 13, diagnosis of schizophrenia	After birth, intermittent response to treatment and acceptance of child	n.a	No menses, no fetal movement, weight gain misattributed by patient	Early in pregnancy	Psychiatric Condition
Narlesky et al. [17]	23	2	No	Stimulant use disorder, schizophrenia	Intermittent denial	*Trauma*: Sexual abuse by brother	n.a	At 6 weeks of gestation	Psychiatric Condition, Early Trauma
Sablone et al. [18]	17	1	No	Borderline personality disorder and intellectual disability	Poor coping skills	n.a	Vaginal spotting, weight gain, no fetal movement	At birth	Psychiatric Condition
	22	2	No	Borderline personality disorder and intellectual disability	Cognitive superficial awareness, affective denial	*Stressor*: unexpected birth of first child	No weight gain	Early in pregnancy	Psychiatric Condition
Schultz and Bushati [15]	26	1	No	Undiagnosed mental illness	n.a	n.a	Normal menses	At 24 weeks by GP (intrauterine death)	Psychiatric Condition
	29	2	No	Undiagnosed mental illness	Continuing denial of first pregnancy	n.a	Normal menses, no fetal movement, misattributed weight gain	Eclamptic seizure at 36 weeks of gestation	Psychiatric Condition
Jimenez et al. [21]	39	8	Married	Trichotillomania, feeling depressed after some childbirths	n.a	*Stressor*: Teenage daughter with leukemia *trauma:* unspecified history	Generally irregular menstrual cycle, no weight changes	At birth	Insufficient signs of pregnancy, early Trauma
Nto-Ezimah et al. [25]	19	1	No	None	Preexisting overweight; living with strict parents	n.a	Regular menses, light nausea, weight gain	At birth	Unconscious defense mechanism
Sar et al. [26]	21	1	Stable relationship	None	Major change in living situation, recently stopped taking oral contraception	*Stressor*: Death of grandfather 6 months prior	2 times light vaginal bleeding, slight weight gain, light nausea	At birth	Unconscious defense mechanism
Chechko et al. [28]	38	3	Married	None	Low emotional awareness	None	Oral contraceptive pill, regular (withdrawal) bleeding, no fetal movement, only slight weight gain	At birth	Insufficient signs of pregnancy
Struye et al. [27]	44	7	Divorced, new relationship for 2 years	None	Restrictive upbringing, no sex-education	*Stressor*: Death of ex-husband shortly before (acute trauma)	Irregular vaginal bleeding, only slight weight gain, no fetal movement, no nausea	At birth	Insufficient signs of pregnancy
Di Giacomo et al. [29]	25	2	Married	None	n.a	None	Oral contraceptive pill, no weight gain, no body changes, no fetal movements	At birth	Insufficient signs of pregnancy
Nanjundaswamy et al. [30]	27	1	Married	None	Premorbid personality, agreed to become parents later	None	Irregular vaginal spotting, some weight gain	At birth	Insufficient signs of pregnancy

*n.a.* not available, *GP* general practitioner

### Denial Related to a Psychiatric Condition

In the past decade, five case reports were published describing women with pregnancy denial likely due to a preexisting mental disorder (see Table 1) [9, 15–18]. Of these, three cases involved psychotic denial, each one of which had a diagnosis of schizophrenia before the onset of pregnancy [16, 17], or a novel psychotic episode in the context of a pre-existing mental disorder [9]. Of interest is the case of one of these women who had a history of pseudocyesis (false pregnancy) [17], a condition characterized by her belief that she was pregnant despite evidence to the contrary [19]. Two women denied their pregnancy twice: one with an unspecified mental disorder (aged 26 and 29 years) [15] and the other with an intellectual disability and additional borderline personality (aged 17 and 22 years) [18].

Given the underlying psychiatric condition, it is difficult to determine how many of these women were truly unaware of their pregnancy. Reality displacement occurs during psychotic symptoms associated with schizophrenia and depression. A misinterpretation and misjudgment of reality may result in the denial of pregnancy in one moment and a likely acceptance of it in the next. Therefore, it is difficult to determine whether pregnancy denial is an independent entity existing alongside a psychiatric disorder, or if it is another symptom of the same.

While pregnancy denial undoubtedly challenges the psychological functioning of mothers, in general, psychiatric disorders have been found to be linked to only a small number of cases. Thus, it seems not to be a primary feature of pregnancy denial, which can occur without any clinical manifestation of psychiatric disorder [2, 13••].

### Denial Related to (Early) Trauma

Being pregnant can trigger the return of traumatic memories, causing the mind to repress the somatic cues of pregnancy [11]. Dissociative symptoms have a close link to trauma, playing a key role during and after labor and delivery [20•]. The trauma can ensue from an early attachment trauma, a history of physical or sexual abuse, as well as trauma associated with the conception of the denied pregnancy, or a combination thereof [1, 11, 20•].

Narlesky et al. [17] described a woman with preexisting schizophrenia and history of pseudocyesis who, as a child, had been sexually abused by her brother (see Table 1). In the case described by Jimenez et al. [21], the woman experienced a tumultuous upbringing marked by an alcoholic father and violence between the parents. Even though the patient denied a trauma history, the authors attributed her denial of pregnancy to her family history and avoidant behavior [21]. None of the other case reports published in the last 10 years reported traumatic life events.

### Denial Related to a Conflict Situation, Denial as an Unconscious Defense Mechanism, and the Benefits of Assumed Non-pregnancy

Based on the notion of parent–child conflict [22], pregnancy can be viewed as a conflict between the mother and the fetus, involving the fetus’ demands and the mother’s willingness to care, with both trying to protect their individual interests [23•]. Severe external stress and internal conflict may cause a woman to use inappropriate defense mechanisms and not accept the underlying reality of her pregnancy. From the psychodynamic point of view, denial may also be seen as a repression mechanism with unconscious blocking of unpleasant emotions, impulses, memories, and thoughts from the conscious mind [24]. In an unchangeable situation [8], a woman’s subconscious decision in favor of her interests, to the detriment of those of the child [23•], may be an emotion-focused survival strategy.

We identified two case reports in which the protection of individual interests was the likely reason behind pregnancy denial (see Table 1) [25, 26]. The women did not recognize they were pregnant until the onset of labor/delivery. One, who was 19 years of age, admitted that pregnancy would have been catastrophic for her, potentially forcing her to commit suicide, if she had discovered the pregnancy before delivery [25]. The other, a 21-year-old woman, was in an emotionally stressful situation after the death of her grandfather and was also preparing for a major change in her life situation by leaving her social environment through a student exchange program [26]. From a psychoanalytical point of view, it may be safe to assume that repression mechanisms likely played a role in both cases.

### Denial Related to the Missed Sign of Being Pregnant

Not recognizing one’s own pregnancy can be due to insufficient cues. While it is not scientifically proved, there is anecdotal evidence of midwives hearing women say that they “didn’t realize” they were pregnant. In most of these cases, there seem to be no underlying psychological or psychiatric problems. In three of the reported cases that fit into this category, the women were 38 years and older and already had multiple children (one woman with eight children, one with three, and one with seven children; see Table 1) [21, 27, 28]. Only in one case, the woman was 25 years old, but already had a child [29], and one case involved a 28-year-old primiparous woman (see Table 1) [30]. The last two of these cases, and the one described by Chechko et al. [28] involved women with stable socioeconomic backgrounds, stable partnerships, and no exceptional life situations. Nanjundaswamy et al. [30] described a woman exhibiting little empathy and problems with emotion regulation, while the one described by Chechko et al. [28] showed decreased self-awareness and an inability to recognize her own feelings. In contrast, Jimenez et al. [21] and Struye et al. [27] presented women with more challenging backgrounds. The case described by Jimenez et al. [21] involved a woman who visited her daughter with leukemia in the hospital, had frequent involvements with child welfare services (with seven children), and was in a distant relationship with her husband who accused her of extramarital affairs. In addition, she had suffered from trichotillomania for 20 years, which, however, appeared to have been unproblematic for her. The case reported by Struye et al. [27] involved a woman currently living with her intimate partner and three children, while three of her other children had been placed for adoption. The woman had grown up in a strictly conservative home where the subjects of sex and sexuality were taboo. She was also described as a person who tended to ignore and forget problems, hoping they would resolve themselves. Thus, in these cases, the women had an elevated threshold with respect to the recognition of emotional and possibly also body-related cues, resulting in a generally reduced self-awareness.

## Discussion

The literature search yielded 12 cases covering women who denied their pregnancies with various background characteristics (see Table 1). In five cases, the women had an underlying mental disorder (of which four had psychotic symptoms and one reported trauma in childhood); in two cases, the women had short-term benefits, given their circumstances, from perceiving themselves as not pregnant. In those cases, the women exhibited a general tendency toward using denial as a defense mechanism, which had been facilitated by the lack of physical symptoms. In the remaining five cases, the women appeared to be utterly convinced that they could not be pregnant, and thus did not experience the signs or symptoms of pregnancy. Although the pregnancy denial cases belonging to this category are more common than others, most of them do not get published, presumably because they are not spectacular enough in comparison to those in which psychiatric condition or trauma play a role. Since it is a fairly common phenomenon, we are of the opinion that the highest priority should be given to determining why healthy women frequently living with their partners deny their pregnancies.

Recognizing pregnancy usually involves noticing the symptoms associated with early pregnancy (e.g., absence of periods, tender, swollen breasts, nausea with or without vomiting, increased urination, and fatigue). However, with the exception of amenorrhea, none of these symptoms is a definite sign of pregnancy, which means that women with irregular menstruation, or those who use contraceptives or have bleeding during pregnancy, are more prone to overlooking the ambiguous symptoms and, thus, not recognizing the state of being pregnant. In the prospective case–control study by Delong et al. [13••], most denied pregnancies occurred while using a contraceptive method, mainly an oral contraceptive. An association between oral contraceptive and pregnancy denial has been described in a number of cases [26, 28, 29]. In denied pregnancies, the physiological symptoms (nausea, amenorrhea, increased breast size, abdomen swelling, weight gain) are often either absent or greatly reduced [3, 13••, 14], which was the case in most of the reports here [21, 27–29]. But do pregnant women have to have symptoms that clearly indicate that they are pregnant? In fact, none of the early pregnancy symptoms (e.g., nausea or vomiting, swollen breasts) is obligatory even though each one is clearly linked to hormonal fluctuations [31]. In general, the sensitivity to hormonal fluctuations is highly individualized and has been found to depend on a number of personal and biological factors, with some women showing both physical and emotional responses to endocrine changes (e.g., premenstrual syndrome or postpartum baby blues) and others showing no response at all [32]. Overall, previous studies have suggested that women with insufficient cues are more likely to be unaware that they are pregnant. According to Delong et al. [13••], pregnancy denial may occur in a particular life circumstance in which pregnancy may not be consciously perceived by some women. Women with pregnancy denial may also have a different perceptual threshold with respect to pregnancy symptoms: They are not focused on getting pregnant because they often believe that pregnancy is not possible [13••]. Because of their conviction that they cannot, or are not allowed to, become pregnant (as in the cases described by Nto-Ezimah et al. [25] and Şar et al. [26]), women often do not pay attention to the signs of pregnancy. These women may also have a tendency toward paying less attention to bodily symptoms in general and have a decreased sense of self-awareness in terms of their own feelings [28]. It is often impossibly difficult to explain why and how pregnancy remains unrecognized [28]. The concept of “somatic denial” may shed some light on this phenomenon. The so-called silhouette effect, characterizing the absence of abdominal swelling [33], suggests a possible somatic denial of pregnancy, the absence of a typical pregnant belly hiding the fact of pregnancy from consciousness [18, 26]. Also, menstruation-like bleeding is frequently reported in denied pregnancy [15, 18, 21, 25, 27, 28]. Nausea is found to be suffered most rarely by women who are unaware of pregnancy until delivery [2].

The underlying mechanisms, however, are not yet understood. The frequently reported correlation of human chorionic gonadotropin (hCG) with nausea and vomiting suggests that women affected by pregnancy denial have lower levels of hCG [20•, 23•, 19]. However, there is a lack of solid scientific data to support this assumption. As it is, even the relationship between hormonal status and vomiting/nausea is debatable [31].

On the other hand, during pregnancy, the physiological and homeostatic mechanisms of the female body change to meet the needs of the fetus [34]. For most women, these changes make it impossible for them to not notice their pregnancy. The experience of pregnancy-related symptoms may also have adaptive functions, likely serving to protect the fetus. For example, morning sickness may prevent a pregnant woman from ingesting foods that may be harmful to her or the fetus at a time when its organ systems are developing [35]. Considering pregnancy as an evolutionary strategy with costs and benefits for parents and offspring, the relevant symptoms are based on maternal costs, benefitting the offspring [36]. Thus, pregnancy denial may occur when it is more beneficial for a woman to perceive herself as not pregnant and therefore not experience the symptoms, a decision albeit made subconsciously. While denied pregnancies are sometimes associated with harmful side effects for the fetus (e.g., low birth weight, prematurity), the mother benefits greatly by incurring only a fraction of the costs normally associated with pregnancy [23•].

Viewed from an evolutionary perspective, this phenomenon may also be seen as an adaptive mechanism, which forces cooperation between mother and fetus under circumstances that may be stressful or threatening. In this regard, the fetus also profits with the mother reducing her investment to ensure survival and the accomplishment of delivery [23•]. In this context, drawing a parallel between the human and animal worlds may be of interest. While in animals there is no pregnancy denial per se, infanticide can be seen as a precursor to it. In mammals, infanticide by females is common when conditions are harsh, and it is particularly costly to have offspring. Thus, infanticide may enable females to derive significant benefits by helping reduce their costs [37].

Furthermore, psychological factors in relation to the recognition of pregnancy seem to play an important role. The likelihood of pseudocyesis, or false pregnancy, is a demonstration of this fact. False pregnancy is a condition in which, despite all the physical signs and symptoms of pregnancy, no child is carried [38]. Women with pseudocyesis have not only been seen to have altered neuroendocrine mechanisms, causing amenorrhea, and abdominal swelling [19], some have even reported feeling fetal movements [19]. The reason behind this is that, in some cases, the body simulates pregnancy as a way of coping. For instance, false pregnancy has been seen in women who desperately want to become pregnant, or those who mourn the loss of reproductive ability [19, 39]. A comparison between the characteristics of false and denied pregnancies has revealed that, with few exceptions, most women are between 20 and 40 years of age, married or are in stable relationships, have had previous pregnancies, and are in relatively good psychological health [20•, 40]. Thus, false pregnancy and pregnancy denial, despite opposite outcomes, have enough in common to represent the two ends of the spectrum of pregnancy-related delusions [20•]. The case described by Narlseky et al. [17] involves a woman with a psychotic pregnancy denial as well as a history of pseudocyesis, indicating that the two phenomena may be part of the same continuum.

Interestingly, nonpsychotic women who are confident they are not pregnant manage to convince their family members, and even their sexual partners, that they are not pregnant [25, 28, 30]. In some cases, as the ones described by Nanjundasmay et al. [30], Nto-Ezimah et al. [25], and Chechko et al. [28], even treating physicians have been reported to attribute complaints and symptoms to abdominal pain or other somatic problems [8, 41]. That even partners can deny having suspected pregnancy suggests that others’ perception of pregnancy is strongly linked to the women’s behavior. Thus, women are perceived as pregnant only when they “signal” this to the environment. This decision can be made by a woman subconsciously, in favor of their benefits [36], based on the demands of their situation. On the other hand, some women seem not to notice the symptoms due to their diminished capacity of self-awareness. In this context, the concept of “somatic denial” should be explored further. Finally, a somewhat diffuse interface between conscious coping strategies and unconscious defense mechanisms appears to underlie the individual dynamics in pregnancy denial [14]. Given the large number of factors (including physiological factors) that contribute to the condition, it is impossible to explain the phenomenon in all its complexity. Women with pregnancy denial have been found to belong to a heterogeneous constituency, precluding any clear explanation as to why they deny their pregnancy or what type of women may be prone to pregnancy denial. Thus, the reported cases cannot be condensed into a simple classification and explained by means of a single psychological model.

## Conclusion

According to the literature, pregnancy denial is a pathological issue, a likely consequence of some trauma, the wish to not have a child (defense mechanism possibly triggered by trauma), or a psychiatric problem of a different origin. However, given that the majority of cases cannot be clearly linked to any of the above reasons, we argue that the phenomenon cannot be put in any particular category as it is not always associated with mental or physiological problems. Under certain circumstances, it can affect any woman. Therefore, it is important for gynecologists as well as general practitioners to be more aware of this phenomenon so that they can conduct appropriate investigations when pregnancies are suspected by them, despite the patients’ categorical denial of the fact.

## Data Availability

Data sharing is not applicable to this article as no new data were created or analyzed in this study.
